# Beyond genomics: using RNA-seq from dried blood spots to unlock the clinical relevance of splicing variation in a diagnostic setting

**DOI:** 10.1038/s41431-025-01792-2

**Published:** 2025-01-28

**Authors:** Aida M. Bertoli-Avella, Mandy Radefeldt, Ruslan Al-Ali, Luba M. Pardo, Sabrina Lemke, Anika Leubauer, Daniel L. Polla, Rebecca Hörnicke, Ligia S. Almeida, Krishna Kumar Kandaswamy, Christian Beetz, Jorge Pinto Basto, Peter Bauer

**Affiliations:** CENTOGENE GmbH, Rostock, Germany

**Keywords:** Medical research, Clinical genetics

## Abstract

We aimed to assess the impact of splicing variants reported in our laboratory to gain insight into their clinical relevance. A total of 108 consecutive individuals, for whom 113 splicing variants had been reported, were selected for RNA-sequencing (RNA-seq), considering the gene expression in blood. A protocol was developed to perform RNA extraction and sequencing using the same sample (dried blood spots, DBS) provided for the DNA analysis, including library preparation and bioinformatic pipeline analysis. Splicing in genes of interest was inspected using IGV, with at least three unaffected individuals as controls. From the 113 variants, we confirmed an abnormal splicing in 64 variants (57%). In 15 variants (13%), we did not observe a splicing alteration. In the remaining 34 variants, no decision could be made on the splicing effect due to insufficient sample quality (21%) or a low number of reads (9%). The most common event leading to aberrant splicing was exon skipping, identified in 31 variants (48%). Other events included cryptic donor/acceptor site usage (*n* = 25; 39%), intron retention (*n* = 4; 6%), and other complex events (*n* = 4; 6%). In three patients, pathologically reduced enzymatic activity (measured using the same DBS) served as additional confirmation of the abnormal splicing caused by variants in *HEXA, GAA*, and *GLA*. We implemented an RNA-seq pipeline using the same sample provided for genomic testing. This multiomic approach, as implemented in our routine diagnostic processes, clarifies the clinical relevance of most of the analyzed variants and delivers more comprehensive genetic testing.

## Introduction

Clinical exome and genome sequencing are proven first-line diagnostic methods for rare diseases with diagnostic yields of up to 60% [1–4]. However, many patients remain undiagnosed, with variants of unknown significance (VUS) or no relevant variants detected even after genome sequencing [1, 4, 5]. In many cases, the interpretation of the clinical significance of the identified variants is challenging due to the lack of knowledge regarding the effect of noncoding variants. These are usually classified as VUS with the assessment of their pathogenicity mainly relying on their rarity and computational predictions. Until now, clinical laboratories have faced difficulties in experimentally assessing the clinical relevance of such variants. One of the main limitations is the lack of biological material to isolate RNA and perform pertinent experiments. Other limitations are related to the lack of sufficiently trained scientists (understaffed laboratories) and streamlined workflows.

Recently, RNA-seq has been implemented as an effective complementary tool in genetic diagnosis [6, 7]. Transcriptome analysis has been used by diagnostic laboratories using RNA extracted from a newly collected blood sample or cultured fibroblast (after skin biopsy) in patients with certain disease groups [8] and no genetic diagnosis after genomic testing. Preliminary work indicates that up to 30% of the VUS could be reclassified based on the results of RNA-seq [9]. In addition, an increase in diagnostic yield of 8-12% has been reported by using RNA-seq as a complementary diagnostic method [6, 7, 10, 11].

So far, most of the published studies have used RNA transcriptome analysis from samples derived from blood, cultured fibroblasts, or biopsies where the quality of RNA is expected to be high when sampling instructions and logistic requirements such as a cool chain are diligently followed. Although preferable, it is not always possible to obtain high-quality RNA for example in resource-limited clinical settings. Therefore, other methods to collect patient material to extract RNA are needed. Within this project, we implemented transcriptome analysis using dried blood spots (DBS) from the same sample from which DNA was previously isolated for genomic testing in our clinical diagnostic laboratory. We focused on patients for whom genomic testing resulted in medical reporting of noncoding variants with unclear/unknown effects. Our results show that RNA-seq analysis for diagnostic purposes from DBS-derived RNA is a helpful tool in the multiomic approach towards the improved assessment of the clinical relevance of noncoding variants.

## Material and methods

### Patients

The current study has been conducted within a diagnostic setting. Written informed consent for genetic testing related to the disease(s) of the patient was obtained. Additionally, the consent form declaration included information regarding the storage of the data and further data processing for research purposes. Written informed consent was given by patients, legal guardians, or referring physicians. The informed consent form is available in English at https://www.centogene.com/downloads.html.

DNA was extracted using standard methods, usually from DBS submitted on filter cards (CentoCard^®^). Next-generation sequencing (panel, exome sequencing, genome sequencing), as well as enzyme activity measurements and biomarker quantification, were requested and performed within our diagnostic setting [1, 3, 12].

### Panel, exome sequencing (ES) and genome sequencing (GS)

For the NGS gene panels, the coding regions, 10 bp of flanking intronic sequences, and known pathogenic/likely pathogenic variants (coding and noncoding) of the selected genes were targeted for analysis [12]. ES/GS were performed as previously described [1, 3]. An in-house bioinformatics pipeline, including read alignment to GRCh37/hg19 genome assembly and revised Cambridge Reference Sequence (rCRS) of the Human Mitochondrial DNA (NC_012920), variant calling, annotation, and comprehensive variant filtering was applied. All variants with minor allele frequency (MAF) of less than 1% in the gnomAD database and disease-causing variants reported in HGMD®, ClinVar, or CENTOGENE’s Biodatabank were evaluated. The investigation for relevant variants is focused on (but not limited to) genes with clear gene-phenotype evidence (based on OMIM® information). All modes of inheritance are considered. If potentially relevant variants are identified in genes of uncertain significance, the ClinGen Clinical Validity Framework for the evaluation of gene-disease relationship was applied [13]. Variants were reported according to the level of evidence as previously implemented in our laboratory [14].

Reported genetic variants were classified according to the published ACMG/AMP guidelines as pathogenic (P), likely pathogenic (LP), and variant of unknown significance (VUS) [15, 16]. Specific recommendations for the application of the PVS1 criterion [17] and the evaluation of the predicted and observed impact on splicing evidence were applied [18]. P/LP variants are considered disease-causing for the specific condition and mode of inheritance. Interpretation of the findings was performed in the clinical context and considering the results from the biomarker testing.

After the genetic diagnosis process concluded, samples from patients with noncoding variants reported were selected for RNA-seq considering that: (i) consent for research analysis was given, (ii) a viable sample was available (DBS not older than 14 days from the blood draw), (iii) sufficient blood expression of the gene of interest.

### Selection of variants and genes for RNA-seq experiments

A total of 108 consecutive patients for whom splicing variants were reported after gene panels, or ES/GS were selected for RNA-seq, considering the gene expression in blood and sample availability. In 67 of the 108 patients, the reported variant was related to the disease and the main clinical question/concern. The other patients were heterozygote carriers of variants with possible aberrant splicing and located in genes related to early-onset diseases. A protocol was developed to perform RNA-seq using the same filter card (DBS) provided for the DNA analysis. The card age (time between blood spotting and DBS storage in our laboratory) was recorded. RNA-seq results were inspected using IGV focusing on the relevant reported variants and compared with at least three unaffected individuals as controls.

### RNA extraction

CentoCards® for all ES/GS cases were split (for DNA and RNA-based sequencing) upon arrival at CENTOGENE and stored at –80 °C to prevent further RNA degradation. Blood collection and sample preparation (spotting on filter card) within 2 h after collection were recommended (with drying at room temperature) before standard shipping. Card age was calculated as the difference between the date of the sample’s arrival/storage and the date of the sample’s collection. Two full spots of the filter card were cut out and transferred into a 2 ml Eppendorf tube. RNA extraction was performed with the Zymo Quick-RNA Miniprep Kit (Zymo Research, Irvine, CA) using additional components and an in-house developed extraction protocol. The RNA integrity number (RIN) of the isolated RNA was assessed with the Agilent 4200 Tapestation System using the RNA High Sensitivity screentape reagents (Agilent, Santa Clara, CA). The quantity of the RNA was measured with the Qubit RNA High Sensitivity Assay (ThermoFisher, Waltham, MA). The RNA from blood kept in PAX gene tubes was extracted with the PAXgene Blood RNA kit (QIAGEN, Hilden, Germany) according to the manufacturer protocol (control samples).

### Library preparation

50 ng total RNA per patient were used as input for the RNA libraries. In a first step, poly(A) mRNA was isolated from the total RNA samples using oligo(dT) magnetic beads (Poly(A) RNA Selection Kit, Lexogen, Vienna, Austria). In a second step, the enriched mRNA was further processed using the Watchmaker Polaris Depletion Kit (Watchmaker Genomics, Boulder, CO) to deplete globin mRNA. The following RNA library preparation steps were carried out with the Watchmaker RNA Library Prep Kit according to the manufacturer’s protocol, with minor modifications. Library preparation conditions, such as fragmentation time and number of PCR-cycles, were optimized to improve data output in terms of library complexity and duplication rate. The amplified libraries were purified using AMPure XP beads (Beckman Coulter, Brea, CA), and their quality and concentration were assessed using an Agilent 4200 Tapestation System with the DNA 1000 Screentape reagents. Final RNA libraries were pooled in equimolar concentrations for sequencing.

### Sequencing

Final pools were sequenced either on an Illumina NextSeq 500 using the NextSeq 500/550 High Output v2.5 reagent or on an Illumina NovaSeq 6000 S4 V1.5 reagents. Samples were run using the 150 bp paired-end protocol. For the confirmation of cases, the already prepared libraries were re-sequenced under the same conditions.

### Bioinformatic analysis

RNA-seq raw data was aligned using two-pass mode with STAR v.2.7.6a against the hg19 reference genome. The read groups were fixed, and the duplicates were marked using Picard tools v.2.23.8 (http://broadinstitute.github.io/picard). Counting the reads was performed by featureCounts/subread v.2.0.1, and normalized expression levels (TPM, RPKM) were calculated with StringTie v2.1.4.

### Statistical analysis

Descriptive statistics of the RNA quality and other metrics for Quality Control of the sequenced genes was presented as median (interquantile range) and proportions for categorical data. Correlations were calculated using Pearson’s correlation function (package ggcorrplot) in R statistical programming language version 4.3.1.

## Results

We selected 113 consecutively reported variants predicted to affect splicing, which were identified in our laboratory following ES/GS or gene panel sequencing, from 108 samples. Table 1 presents the main features of the patient’s samples and the variants selected. Most of the samples (59%) had a card age of up to 7 days and 42% had a card age between 8 and 15 days. The samples came mainly from the Middle Eastern/North African regions (63%), and Latin America (18%), among others (Table 1). Of the 113 variants tested, most were heterozygous (72%) and located in noncoding regions that did not affect the canonical splicing sites (up to 824 base pairs, 57%).

**Table 1:** The main features of the samples (*n* = 108) and variants (*n* = 113) tested in this study by gene panel, exome, or genome sequencing.

Sample and variant features	Number	Percentage
**Card age in days (*n*** = **108)**		
1–3	18	17
4–7	45	42
8–10	29	27
10–15	16	15
**Gender**		
Male	54	50
Female	54	50
**Origin of the patient samples**		
MENA	68	63
LATAM	19	18
Europe	12	11
South Asia	8	7
South Africa	1	1
**Variant zygosity (*n*** = **113)**		
Hemizygous	6	5
Homozygous	26	23
Heterozygous	81	72
**Variant position**		
Canonical intronic position 1/2	49	43
Other intronic regions	64	57
**Variant type**		
Single nucleotide variant (SNV)	96	85
Small indels	17	15

Card age was calculated as the date of sample received/stored – date of sample collection (as registered in the filter card).

*MENA* Middle East-North Africa, *LATAM* Latin America.

### Quality control of the RNA sequencing

**Fig. 1:**
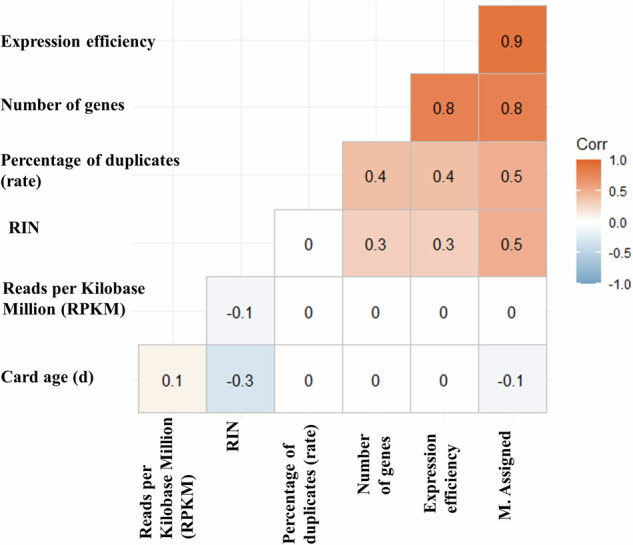
Correlation Matrix of different quality criteria of the RNA sequencing procedure. Figure presents the pairwise correlation between different parameters of RNA quality extraction. The strength of correlations is represented in colors with the red color depicting strong positive correlations and blue negative correlations. Non-significant correlations are depicted as white blocks. Strong positive correlations were observed between average reads per million and the reads assigned to genes. Card age showed weak negative correlations with other parameters of RNA quality. RIN = RNA integrity number; Card age = age [in days] of the filter cards from day of sample extraction until storage at –80 °C (preservation of RNA quality); RPKM = average RPKM values obtained from various RNA sequencing runs from DBS cards (cutoff for inclusion was set to a minimum of RPKM = 4); % Expression efficiency = Ratio of exonic reads to total reads; # Genes = number of genes detected with at least 5 reads; % Assigned = % assigned reads to features (genes); % Dups = % duplicate rate; M Assigned = millions unique assigned reads to features (genes).

The median RIN number of extracted RNA was 3.6 (interquartile range: 2.3–4.9) with a maximum value of 8.3 (one sample). Figure 1 shows a low correlation between RIN number and average RPKM values (Pearson’s correlation coefficient: –0.1; *p* value = 0.3). There was no correlation between RIN and RNA concentration (Pearson’s correlation coefficient: 0.03; *p* value = 0.76) and a weak negative correlation between the card’s age and RIN values (Pearson’s correlation coefficient: –0.3; *p* value = 0.002). The insert size of the obtained reads was approximately 200 bp. The median sequencing depth of the samples was 46 million reads (interquartile range: 33.2-51.6).

**Fig. 2:**
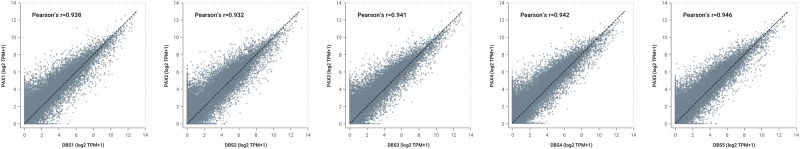
Correlation between expression derived from PAX-tubes and DBS. Scatter plots showing Pearson’s r correlation (0.93–0.95) between gene expression [log2(TPM + 1)] of PAX-tubes and DBS samples for five different individuals (DBS cards at room temperature for two days before storage at –80 °C until further processing). The high correlation between the RNA-seq results from the different sources supports the use of DBS as a sample for RNA-seq.

Figure 2 plots the gene expression levels (log counts) derived from PAX-tubes collected blood against DBS expression values from five individuals. As shown in this figure there was a strong correlation (0.93), with larger variation for lowly expressed genes. The overlap of expressed genes in samples derived from DBS was compared with samples from PAX-tubes for genes at a threshold of TPM ≥ 4 (Supplementary Fig. 1). We found that a high proportion of genes detected in the PAX-RNA samples were also present in the DBS-RNA samples (80.6%). Both sample sources show a high degree of overlap in the detected genes that are related to OMIM disease-related genes, considering that approximately 71% of these genes are expected to be expressed in blood [19].

**Fig. 3:**
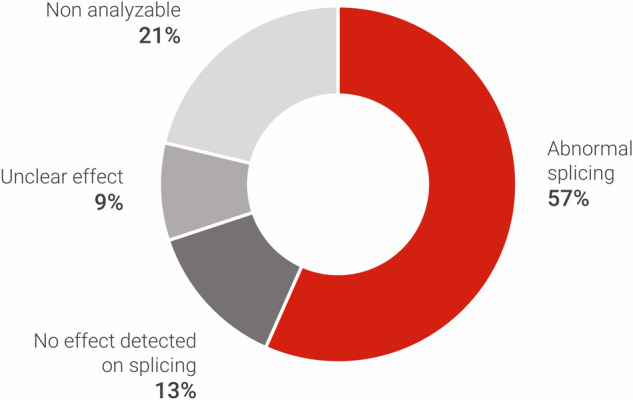
Summary of the results of the RNA-seq analysis of 113 variants. Abnormal splicing was observed in 64 variants (57%) with 30 (47%) not located in canonical splicing sites. Insufficient number of reads was the most common cause leading to non-analyzable/unclear results.

Of the selected variants for this study, 24 could not be analyzed due to either degraded RNA or lower-than-expected expression (21%) (Fig. 3).

### Abnormal splicing patterns in the dataset

**Fig. 4:**
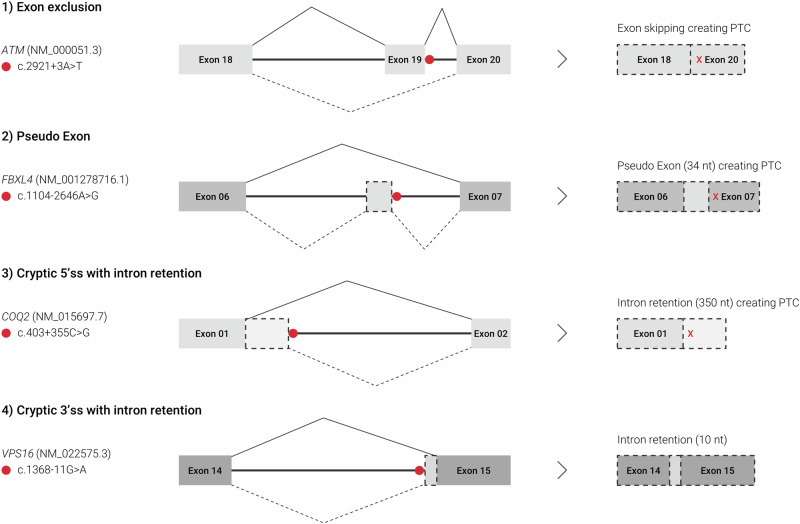
Schematics showing examples of splicing effects detected. (1) Exon exclusion, (2) Pseudo exon, (3) Cryptic donor splice site with intron retention, (4) Cryptic acceptor splice site with intron retention. Left: The solid line shows the regular splicing pattern, and the dashed lines show the observed abnormal splicing. Right: Resulting transcripts with a red cross indicating a premature stop codon. The red dot indicates the location of the noncoding variant. The IGV screenshots from these cases can be found in the Supplementary Fig. 4.

From the 113 reported variants, we confirmed abnormal splicing in 64 variants (57%). In 15 variants (13%) there was no alteration in splicing observed by RNA-seq (Fig. 3). In 34 variants, no decision could be made on the splicing effect because the sample was non-analyzable (21%), or the results were unclear mainly due to an insufficient number of reads (9%) (Fig. 3). Of the 64 variants with a clear abnormal splicing effect, 34 (53%) were affecting the canonical splicing sites, and 30 (47%) were in other non-coding regions. The most common event leading to aberrant splicing was exon skipping, identified in 31 variants (48%). Other events included cryptic donor/acceptor sites (*n* = 25; 39%), intron retention (n = 4; 6%), and other complex events (*n* = 4; 6%) (Fig. 4).

Splicing prediction scores (SpliceAI [20]) were also compared to the obtained RNA-seq results. Of the 64 variants with observed abnormal splicing, 43 (68%) had a SpliceAI score above 0.8 (highly probable to have a splicing effect), and for 21 (32%) variants, the score was below this value. SpliceAI scores are next to each variant in Table 2 and Supplementary Table, allowing the comparison between the predicted and observed splicing effect. Discordantly low Splice AI scores (<0.2) were observed for some variants for which a splicing effect was confirmed via RNA-seq. Most of these variants were far from the canonical splicing sites (for example *GLA*, NM_000169.2:c.801+48 T > G).

**Table 2:** List of VUS detected in 18 patients with abnormal splicing observed after RNA-seq from DBS.

Disorder, MOI	HPO terms	SNV Gene	Reference sequence	SNV nt position	Intron	Variant Zygosity	Effect on mRNA	Splice AI	Splicing effect category	Effect on protein	Variant classification and criteria*
**Primary coenzyme Q10 deficiency type 1, AR**. **Osteogenesis imperfecta type XIV, AR (due to hom. del**. ***TMEM38*)**	Abnormal cerebral white matter morphology; Abnormal CNS myelination; Abnormal facial shape; Abnormality of limb bone morphology; Developmental regression; Failure to thrive; Lactic acidosis; Reduced bone mineral density; Rigidity; Seizure; Spasticity; Ventriculomegaly	*COQ2*	NM_015697.7	c.403+355 C > G	Intron 1	Hom	Cryptic donor splice site with intron retention	0.63	Cryptic 5’ss	PTC (likely NMD)	VUS PM3_supp, PM2_supp, PP3 (PVS1 not applied due to partial effect with low number of normal reads observed)
**Rickets due to defect in vitamin D 25- hydroxylation deficiency, AR**	Elevated circulating alkaline phosphatase concentration; Elevated circulating parathyroid hormone level; Low levels of vitamin D	*CYP2R1*	NM_024514.4	c.1331-1 G > A	Intron 4	Het (with another het LP variant in the same gene)	Cryptic acceptor splice site with intron retention	0.90	Cryptic 3’ss	Partial intron retention, changes the reading frame of the last exon	VUS PVS1_mod (RNA), PM2_supp, PM3, BP5 (hom in a patient in our biodatabank with an alternate molecular etiology)
**Vulto-van Silfout-de Vries syndrome, AD**	Coarse facial features; Cryptorchidism; Macroglossia; Micrognathia; Premature birth; Proptosis; Seizure; Thick vermilion border	*DEAF1*	NM_021008.3	c.1127-2 A > G	Intron 8	Het	Exon 9 skipped, cryptic acceptor splice site with partial exon exclusion	0.99	Exon exclusion, cryptic 3’ss	PTC (likely NMD)	VUS PVS1_mod (RNA), PM2_supp Exon 9 is in-frame and removes less than 10% of the protein
**Intellectual developmental disorder type 7, AD**	Microcephaly, Autistic behavior, Intrauterine growth retardation, Neonatal hypoglycemia, Neurodevelopmental delay	*DYRK1A*	NM_001396.3	c.1098+2 T > C	Intron 7	Het, de novo	Exon 7 skipped	0.91	Exon exclusion	In frame del of 49 aa (exon 8)	Likely Pathogenic (upgraded) PVS1_strong (RNA) In frame but in kinase domain, PM2_supp, PM6_supp (assumed de novo)
**Mitochondrial DNA depletion syndrome type 13, AR**	Abnormal facial shape; Abnormal thumb morphology; Anemia; Arachnoid cyst; Failure to thrive; Frontal bossing; Global developmental delay; Hypotonia; Lactic acidosis; Long philtrum; Low-set ears; Metabolic acidosis; Micrognathia; Microtia; Posteriorly rotated ears; Smooth philtrum; Toe syndactyly; Ventriculomegaly	*FBXL4*	NM_001278716.1	c.1104-2646 A > G	Intron 6	Hom	Pseudoexon	0.95	Pseudoexon	PTC (likely NMD)	VUS PM2_supp, PP3 (splice AI), PM3_supp (two cryptic exons, unclear final effect)
**Fabry disease, XL**	Abnormality of the cardiovascular system	*GLA*	NM_000169.2	c.801+48 T > G	Intron 5	Hem (high biomarker and low enz activity)	Cryptic acceptor splice site with intron retention	0.16**	Cryptic 3’ss	PTC (likely NMD)	Likely Pathogenic (upgraded) PM2_supp, PS4_supp, PS3; PP4
**GM2-gangliosidosis, AR**	Delayed speech and language development; Developmental regression; Generalized-onset seizure; Hyperreflexia; Hypertonia; Hypotonia; Intellectual disability; Neurodegeneration; Peripheral demyelination; Spasticity; Visual impairment	*HEXA*	NM_001318825.1	c.492+3_492+6del	Intron 4	Hom (beta-hexosaminidase A activity is decreased)	Exon 4 skipped	0.48	Exon exclusion	PTC (likely NMD)	Likely Pathogenic (upgraded) PVS1_strong (RNA), PM2_supp, PM3_supp, PP4 (abnormal enzymatic activity)
**Claes-Jensen type of X-linked syndromic intellectual developmental disorder**	Accessory spleen; Paroxysmal drowsiness; Hypertonia; Stereotypical body rocking; Agitation; Autistic behavior; Strabismus; Intrauterine growth retardation; Abnormal facial shape; Global developmental delay; Repetitive compulsive behavior; Low frustration tolerance	*KDM5C*	NM_004187.3	c.2243+5 G > A	Intron 15	Hem	Cryptic donor splice site with intron retention, exon 16 skipped	0.37	Cryptic 5’ss, Exon exclusion	PTC (likely NMD)	VUS PM2_supp, PP3 (PVS1 not applied -partial effect)
**Buschke-Ollendorff syndrome, AD**	Knee pain; Joint swelling; Limited knee flexion/extension; Abnormal bone ossification; Abnormality of femur morphology; Abnormality of tibia morphology	*LEMD3*	NM_014319.4	c.2573-11 T > G	Intron 12	Het	Cryptic acceptor splice site with intron retention	0.94	Cryptic 3’ss	PTC (likely NMD)	VUS PM2_supp, PP3 (splice AI) (MANE NM_014319.4 last exon)
**Schwannomatosis, AD**	Schwannoma; Multiple cafe-au-lait spots; Peripheral Schwannoma	*LZTR1*	NM_006767.3	c.1942+2 T > C	Intron 16	Het	Cryptic donor splice site with partial exon exclusion	0.82	Cryptic 5’ss	PTC (likely NMD)	VUS PVS1_mod (alternate site preserves reading frame), PM2_supp
**Mitochondrial complex I deficiency nuclear type 6, AR**	Strabismus; Dysarthria; Gait disturbance; Neurodegeneration; Lethargy; Tremor	*NDUFS2*	NM_004550.4	c.703-11 T > G	Intron 07	Hom	Exon 8 skipped	0.29	Exon exclusion	In-frame del of 26 aa (exon 8)	VUS PM3, PM2_supp, PP1_supp, exon 8 is in-frame (less than 10% would be removed, and partial effect)
**NF1-related disorder, AD**	Vascular malformation of the lip; Cafe-au-lait spot; Vascular skin abnormality; Capillary hemangioma; Cavernous hemangioma	*NF1*	NM_001042492.2	c.6820-1 G > C	Intron 45	Het	Exon 45 skipped	0.98	Exon exclusion	In-frame exon skipping (exon 45)	VUS PVS1_mod (RNA), PM2_supp, PP4
**Intellectual developmental disorder type 75 with neuropsychiatric features and variant lissencephaly, AR**	Acute lymphoblastic leukemia; Attention deficit hyperactivity disorder; Autistic behavior; Global developmental delay; Intellectual disability; Macrocephaly; Stereotypy	*PIDD1*	NM_145886.3	c.2042-2 A > G	Intron 12	Hom	Intron retention	0.99	Intron retention	PTC (likely NMD)	VUS PVS1_mod (RNA), PM2_supp, PM3_supp (partial intron retention and alt splice site)
**Global developmental delay with speech and behavioral abnormalities, AD**	Autism; Medial flaring of the eyebrow; Widened distal phalanges; Prominent nasal tip; Epicanthus; Poor eye contact; Aggressive behavior; Anxiety; Broad forehead; Synophrys; Broad eyebrow; Almond-shaped palpebral fissure; Long philtrum; Large earlobe; Anteverted ears; Pointed helix; Cryptorchidism; Global developmental delay; Joint laxity; Hypotonia; Talipes valgus	*TNRC6B*	NM_001024843.1	c.46-2 A > G	Intron 3	Het	Cryptic acceptor splice site with intron retention	0.96	Cryptic 3’ss	PTC (likely NMD)	VUS PVS1 is not applicable (in MANE transcript NM_001162501, the variant is located upstream of 5’UTR). BP5 (one het patient in our biodatabank with an alternate molecular etiology), PM2 not applicable (9 het in gnomAD, most reported variants are de novo)
**Dystonia type 30, AD**	Gait disturbance; Dystonia	*VPS16*	NM_022575.3	c.1475 A > G	Intron 14	Het	Exon 15 skipped	0.82	Exon exclusion	PTC (likely NMD)	VUS PM2_supp, PP3 (GDR strong for AD Dystonia, pli 0, no animal model, incomplete penetrance, PVS1 not applied. One additional het patient plus unaffected mother)
**Dystonia type 30, AD**	Dystonia; Torticollis; Limb dystonia; Craniofacial dystonia; Gait disturbance; Neurological speech impairment	*VPS16*	NM_022575.3	c.1368-11 G > A	Intron 23	Het	Cryptic acceptor splice site with intron retention	1.00	Cryptic 3’ss	Inframe insertion of 3 aa	VUS PM2_supp, PS4_supp, PP3 (in two unrelated het patients in our biodatabank, plus an unaffected mother, the alternate site would preserve the frame)
**Dystonia type 30, AD**	Generalized dystonia; Dystonia; Tremor	*VPS16*	NM_022575.3	c.2375+1 G > T	Intron 23	Het	Intron retention, cryptic splice site with partial exon exclusion	0.98	Cryptic 3’ss	PTC (escapes NMD) and partial deletion inframe of exon 23	VUS PM2_supp, PP3 (in region predicted to escape NMD)
**Wiskott-Aldrich syndrome, XL**	Atrial septal defect; Ventricular septal defect; Thrombocytopenia; Recurrent infections	*WAS*	NM_000377.2	c.777+5 G > C	Intron 8	Hem	Exon 8 skipped	0.95	Exon exclusion	PTC (likely NMD)	Likely pathogenic (upgraded) PVS1_strong (RNA- the effect is not complete, 82% aberrant PMID: 15389128), PM2_supp, PS4_supp

*Classification according to our application of the ACMG/AMP ClinGen SVI general recommendations. ** indicates low SpliceAI score (<0.2)

*MOI* mode of inheritance, *SNV* single nucleotide variant, *nt* nucleotide, *AR* autosomal recessive, *AD* autosomal dominant, *XL* X-linked, *Hom* homozygous, *Het* heterozygous, *ss* Splicing site, *aa* amino acid, *del* deletion, *PTC* premature termination codon, *NMD* nonsense-mediated decay.

### Clinically relevant variants

In the patients for whom the reported variant was related to the main clinical question and presenting phenotype, 35 (51%) variants were classified as P/LP, 30 (46%) as VUS, and four were classified as risk factors (*GALT* haplotype). We confirmed abnormal splicing in 46 (70%) of these variants related to the phenotype of the patient (26 P/LP, 17 VUS, and three risk factors, Supplementary Table).

A list of the 18 patients with reported VUS and confirmed abnormal splicing using RNA-seq, their corresponding HPO terms, and the associated Mendelian disorder are presented in Table 2. The classification of four variants was upgraded from VUS to LP.

## Discussion

More than half of the patients for whom ES/GS is performed remain undiagnosed as noted in a recent metanalysis [4]. This can be caused among others by (i) test inability to detect the DNA variation (e.g., noncoding variant not detected by ES), (ii) difficulties in the interpretation of the detected variants e.g., noncoding variant detected by GS but with unknown effect, (iii) variants in genes with unknown function or role in disease.

In most of the recent studies on validation of abnormal splicing based on RNA-seq, whole blood or biopsy material from muscle, skin (fibroblasts) [11] or bone marrow [6] have been used. We showed that extracting RNA from DBS produces enough RNA for sequencing even when the starting quality of RNA is in some samples not optimal. Despite the moderate to low RIN values, we were able to extract enough RNA from DBS for the sequencing and identification of the variants and their splicing effects. This suggests that RIN metrics do not correlate strongly with the amount of RNA input needed for sequencing. RIN is a measure of total RNA integrity that includes different types of RNA, of which mRNA accounts for only 5%. Sonntag et al. showed that RIN does not always predict RNA integrity, especially at lower values of RIN [21]. Although the study was conducted on post-mortem brain tissue, it suggests that other RNA integrity parameters may be needed.

There are several advantages of using the DBS for RNA-seq: (i) RNA-seq can be performed in the same sample provided for DNA sequencing, (ii) reduces the time to reach a diagnosis providing a faster answer to the patient and family, (iii) it spares resources and time dedicated to multiple appointments and re-sampling, (iii) offers advantages in sample logistics, especially for patients living far away from clinics or hospitals.

An important parameter to consider though, is the time elapsed from preparing and sending a DBS card to processing lab time. Our solution included storing the DBS at -80°C on the same arrival day to the laboratory. Our data showed a (weak) negative correlation between card age and RIN, with optimal results obtained with a card age of up to 7 days (Supplementary Fig. 2). A limitation of the technique is that the gene expression levels in RNA from DBS are expected to be lower than those from other biological samples, such as whole blood or tissue biopsies. In this analysis, we showed a high correlation between the level of expression of genes in RNA derived from PAX gene blood with those from DBS (Supplementary Fig. 1), similar to a previous study [22].

Functional data or assays implemented to determine the impact of a genetic variant have been shown to be an excellent tool for the assessment of the clinical impact or relevance of genetic variants. However, variant-centered assays are difficult to implement in routine diagnostic labs. Novel/rare noncoding variants remain classified as VUS, yet, they have the potential to alter splicing, gene expression, or function. Having a high throughput genome-wide approach to systematically assess the relevance of noncoding is then a valuable addition to the diagnostic workflow coupled with ES/GS. Although the utility of RNA-seq extends beyond abnormal splicing detection, to the assessment of gene expression and detection of allelic imbalances [23], we have focused on the former for an initial proof-of-concept design. As we have shown here, for 57% of the investigated (noncoding) variants, abnormal splicing was confirmed supporting the variant causality.

### Confirmation of RNA alterations in well-known pathogenic variants—selected examples

Among the P/LP variants, we identified a patient who presented with chronic hemolytic anemia and had two heterozygous intronic variants in *HBB*. The NM_000518.4:c.92+6 T > C variant is one of the most frequent *HBB* pathogenic variants in the Middle Eastern region [24], the variant was initially reported in 1982 (also known as IVSI-6) [25]. Since then, abnormal splicing of this variant was confirmed in a transgenic mouse model [26] and it was predicted to affect splicing by different in silico approaches [27], but studies in human samples were lacking. The variant has been reported in a multitude of patients and publications (HGMD database) with classifications of P/LP and VUS in ClinVar (Variation ID:15450). We showed that this variant activates a cryptic 5’ splice site leading to an exon exclusion and a partial intron retention. The other *HBB* variant (NM_000518.4:c.315+1 G > A) has been previously reported in patients with autosomal recessive beta-thalassemia (HGMD database) and ClinVar (Variation ID: 15438 – P). It was first reported in 1982 when Treisman et al. cloned the *HBB* gene from a fetus with beta-thalassemia and showed two abnormally spliced *HBB* RNA with partial intron 2 retention and skipping of exon 2 [28]. Our RNA-seq results confirmed these findings, the variant leads to a skipping of exon 2 as well as to intron 2 retention. The diagnosis of autosomal recessive beta thalassemia was confirmed in our patient (Supplementary Figs. 3A and 3B).

In another patient with global developmental delay, hypotonia, and persistent head lag, a homozygous variant NM_000152.3:c.2647-7 G > A was detected in *GAA*. The variant was first reported by Cipullo et al. [29] and it is known in ClinVar (Variation ID: 551106 – P/LP and VUS). Sampaolo et al. [30] studied the variant in muscle tissue from patients and described that the variant created a novel acceptor splice site in intron 18, with the introduction of five nucleotides of the intron 18 in the *GAA* RNA sequence, producing a frameshift and a premature truncation of the protein [30]. Our RNA-seq results in DBS confirmed this finding (novel acceptor splice site in intron 18 with partial intron inclusion, Supplementary Fig. 3C). The latter patient also had pathologically decreased alpha-glucosidase activity, which was measured in the same DBS sample in our laboratory, confirming the diagnosis of Pompe disease.

### Clarifying the effect of VUS—selected examples

In another interesting case, an adult male patient with clinical suspicion of Fabry disease, GS identified a hemizygous variant in *GLA* (NM_000169.2:c.801+48 T > G). The variant was reported previously in two male patients with cardiomyopathy, but RNA studies were not performed [31]. We detected abnormal splicing with the creation of a new splice site and partial intron retention leading to premature termination (Supplementary Fig. 3D). The patient also presented with pathologically decreased alpha-galactosidase activity and increased biomarker, both measured in the same DBS. Taken together, the variant was classified as LP and the diagnosis of X-linked Fabry disease was established in the patient.

Other relevant examples include a patient with global developmental delay and regression, failure to thrive, lactic acidosis, rigidity, and seizures, in whom GS detected a homozygous deep intronic variant in *COQ2* (NM_015697.7:c.403+355 C > G), the variant is extremely rare (not found in gnomAD) and detected for the first time on our laboratory. Through RNA-seq analysis, we detected that the variant created an alternative donor splicing site and partial intron retention. The variant is classified as VUS and reported for autosomal recessive primary coenzyme Q10 deficiency type 1, which clinically overlaps with the phenotype of the patient (Supplementary Fig. 4).

### Final remarks

The use of RNA-seq in combination with ES/GS has been shown to increase the diagnostic yield of these tests, as well as to assist variant classification, providing an unbiased, genome-wide approach [23, 32]. In conclusion, we implemented an RNA-seq pipeline using the same sample (DBS) provided for ES/GS, to deliver more comprehensive genetic tests by exploring the splicing effect of relevant noncoding variants. Our next efforts will focus on the expansion of the diagnostic applications of the RNA-seq. in the routine diagnostic setting for rare diseases.

## Supplementary information

Supplemental Figures

Supplemental material

## Data Availability

The datasets generated and analyzed during the current study are available from the corresponding author upon reasonable request.
