# Supplementary material for: Beyond genomics: using RNA-seq from dried blood spots to unlock the clinical relevance of splicing variation in a diagnostic setting
Source: Eur J Hum Genet. 2025 Jan 28;33(5):614–23. doi: 10.1038/s41431-025-01792-2 (PMC12048715; doi:10.1038/s41431-025-01792-2)

Supplementary Figure 1. Venn diagram showing the intersection of the expression of genes detected after RNA-seq of samples from PAX-tubes and DBS (TPM ≥ 4, median values from five individuals) as well as the overlap with OMIM disease-associated genes. A high proportion of genes detected in the PAX-RNA samples were also present in the DBS-RNA samples (80.6%). Both sample sources show a high degree of overlap in the detected genes that are related to OMIM disease-related genes, considering that approximately 71% of these genes are expected to be expressed in blood (PMID: 31160820). The values (n) refer to the number of genes.

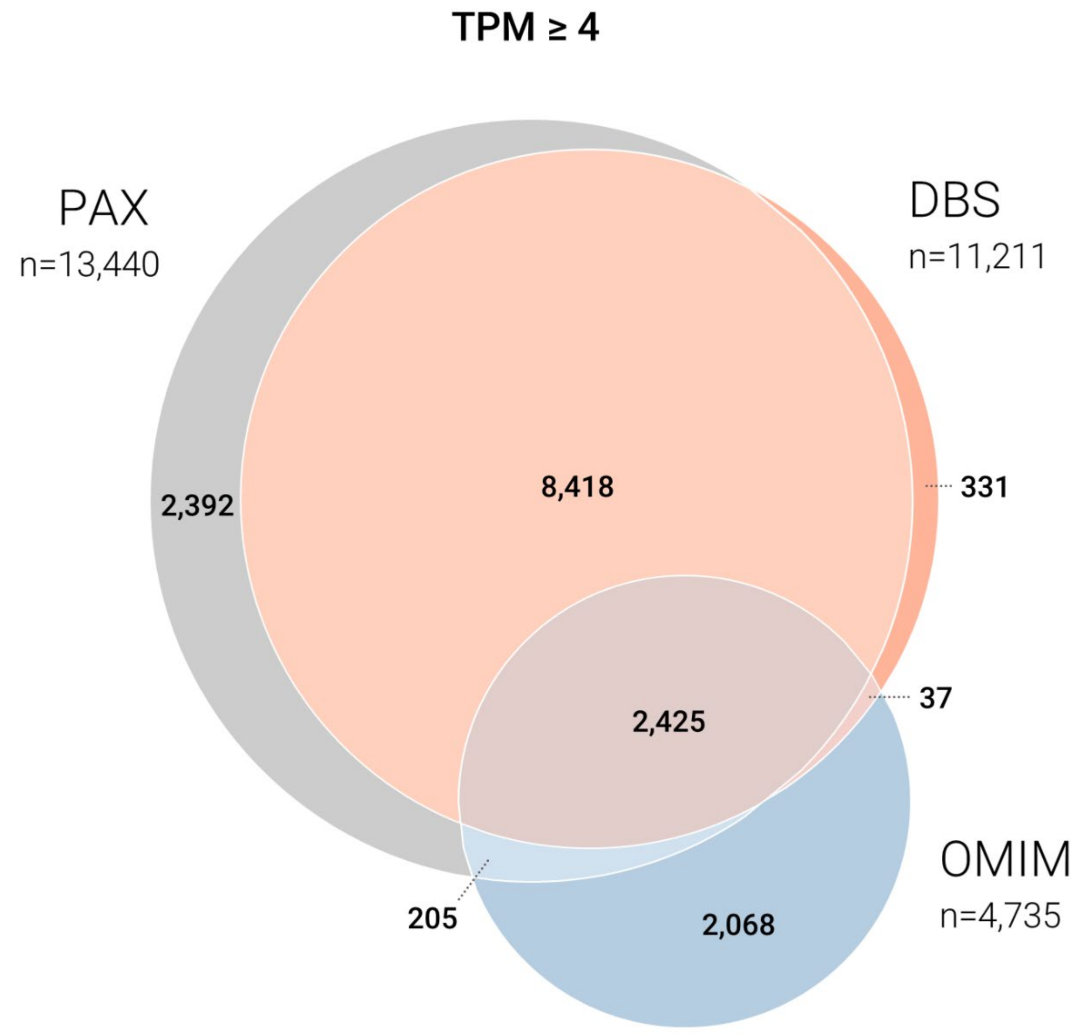

Supplementary Figure 2. Distribution of RNA Integrity Numbers (RIN) shown for RNA extracted from filter cards of different card ages. Fourteen percent of the samples are below the RIN cut-off value of 2.5 with card ages of 8-14 days (red bars)**, w**hereas only 2.9% of the samples with a card age of up to 7 days are below the RIN of 2.5 (grey bars)

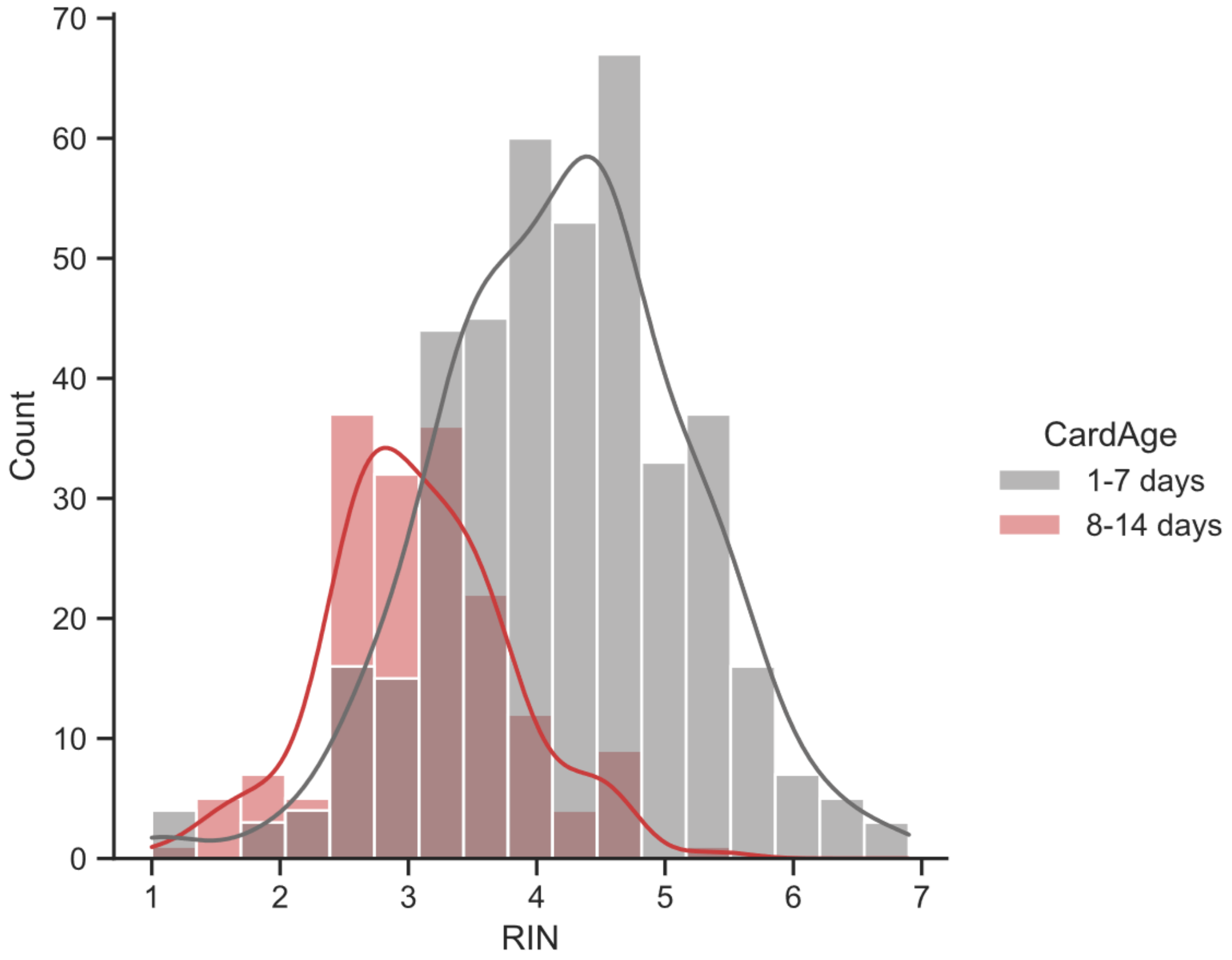

Supplementary Figure 3. Screenshots from the Integrated Genome viewer (IGV) showing splicing alterations.

3A. Patient with heterozygous *HBB* pathogenic NM_000518.4:c.92+6T>C variant which activates a cryptic 5' splice site leading to a 16bp deletion of exon 1 resulting in a premature stop codon with suspected NMD. In addition, the patient was heterozygote for the intronic pathogenic variant NM_000518.4:c.315+1G>A leading to the skipping of exon 2, an activation of a cryptic splice site in exon 2 as well as intron 2 retention. **Left)** Sashimi plot showing the splicing effects in this patient compared to controls **Right)** Alignment tracks from IGV for the same patient. Colored arrows indicate junction reads for exon 1 cryptic splice site (red), exon 2 skipping (blue) and exon **2** cryptic splice site (green). Further confirmation experiments are presented in Supplementary Figure 4.

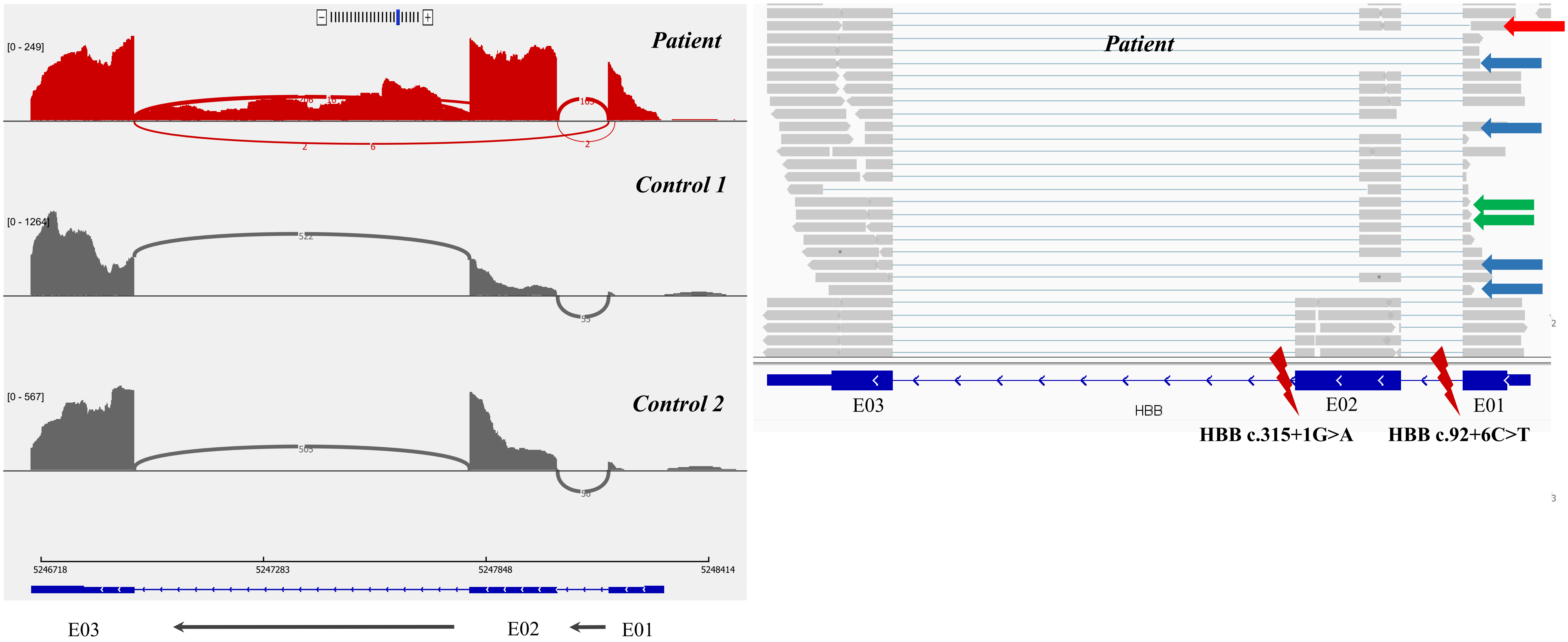

Supplementary Figure 3B. Confirmation of observed *HBB* splicing abnormalities detected by RNA-seq**.** *HBB* relative expression was determined via RT-qPCR in samples heterozygotes for different *HBB* variants compared to three control samples (reference gene: *ACTB).*
A) Exon1/exon3 junction primers were designed to detect skipping of exon2. **B)** Exon1 cryptic splice/exon2 junction primers were used to confirm the cryptic splice site in exon 1.
The samples were measured in duplicates, except for the patient sample (box), because the amount of RNA was limited.

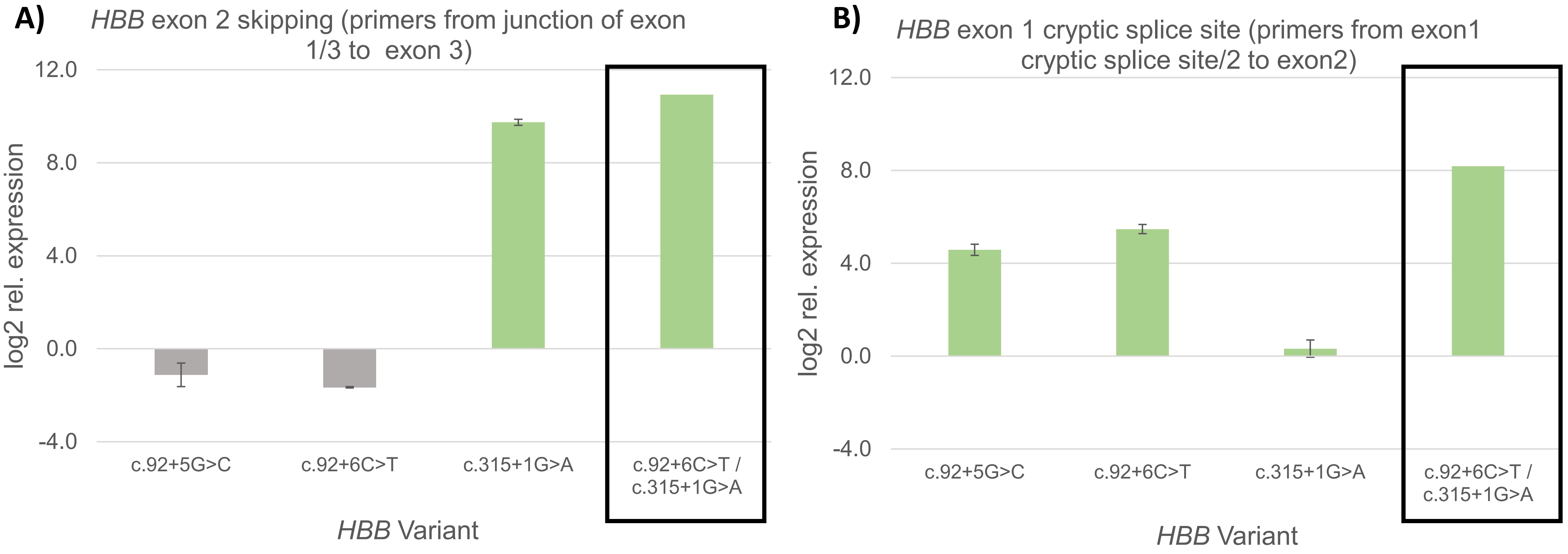

Supplementary Figure 3C: IGV screenshots showing the RNA-seq results from a patient with a homozygous likely pathogenic *GAA* variant NM_000152.3:c.2647-7G>A, leading to a novel acceptor splice site in intron 18 with partial intron inclusion.

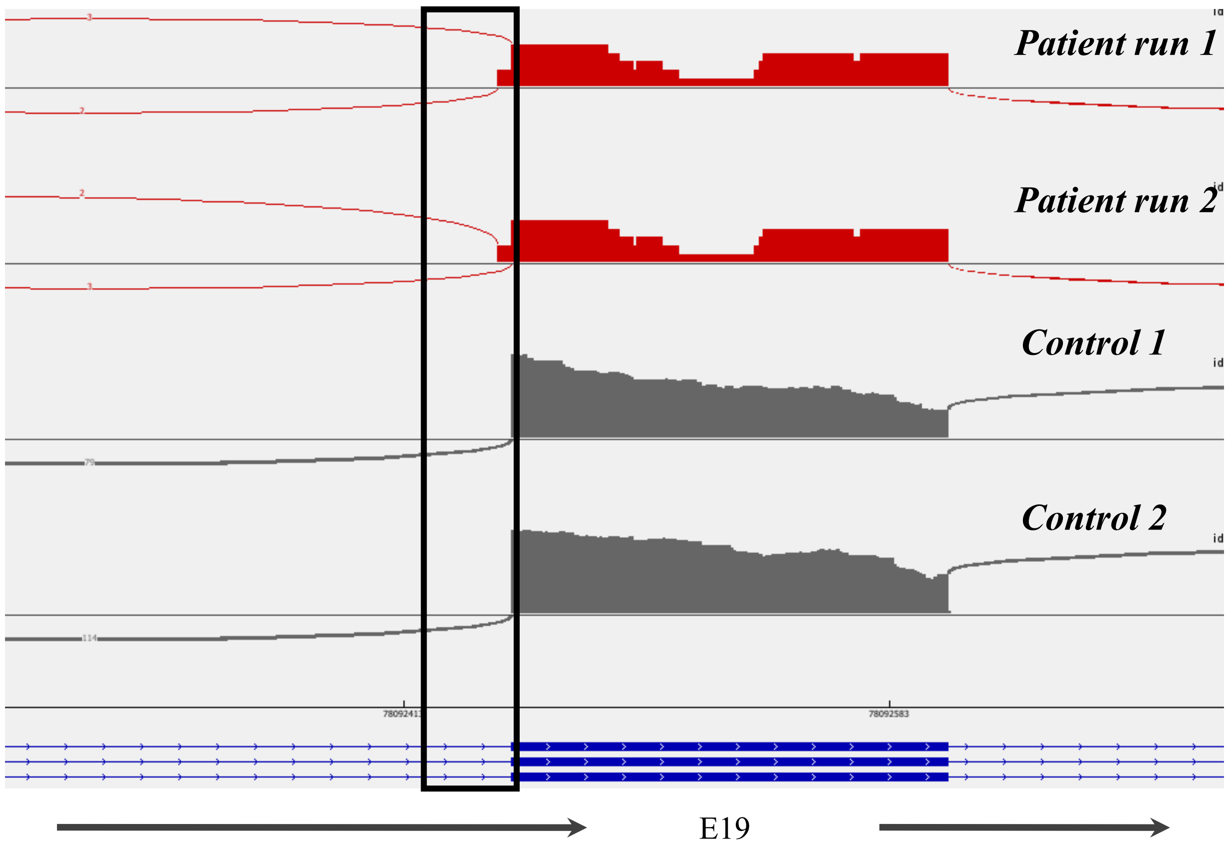

Supplemental figure 3D: IGV screenshots showing the RNAseq-results from a patient with a hemizygous likely pathogenic variant in *GLA* (NM_000169.2:c.801+48T>G) leading to abnormal splicing with creation of a new splice site and partial intron retention and premature protein termination

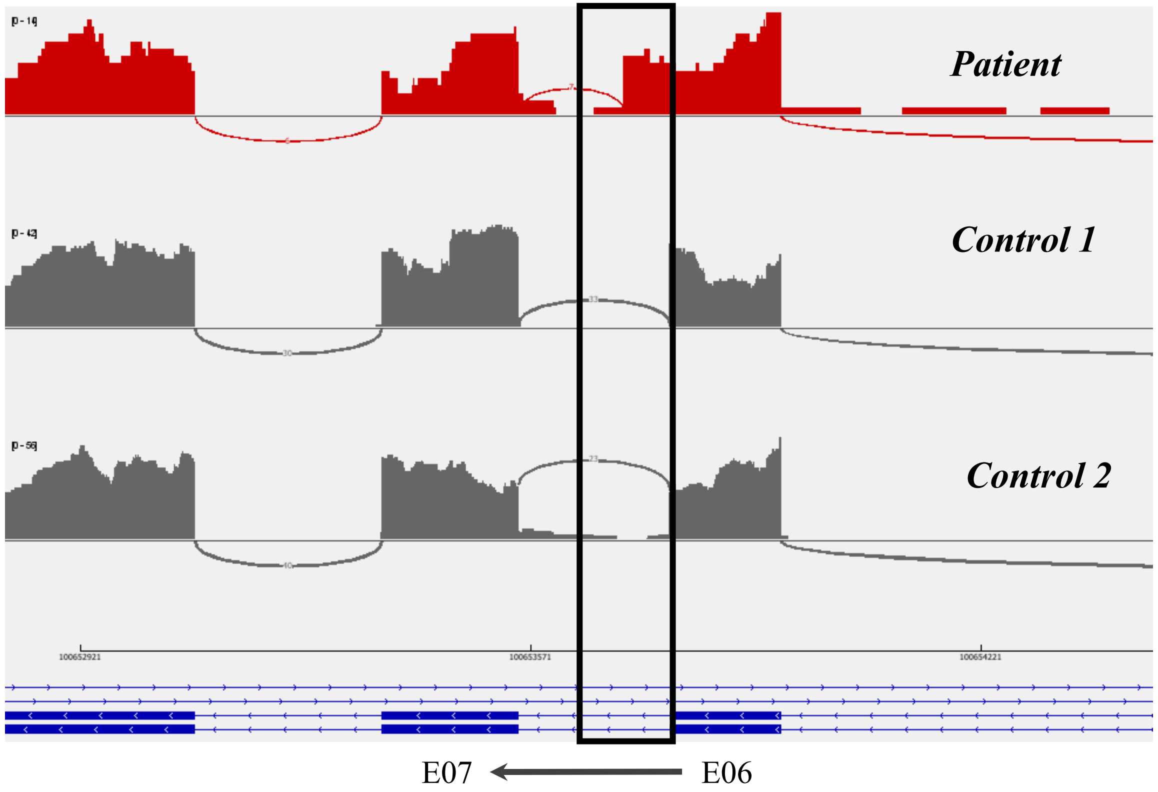

Supplementary Figure 4. Screenshots from the IGV show splicing alterations from the examples from Figure 4.

1) Exon exclusion

***ATM* NM_000051.3:c.2921+3A>T**

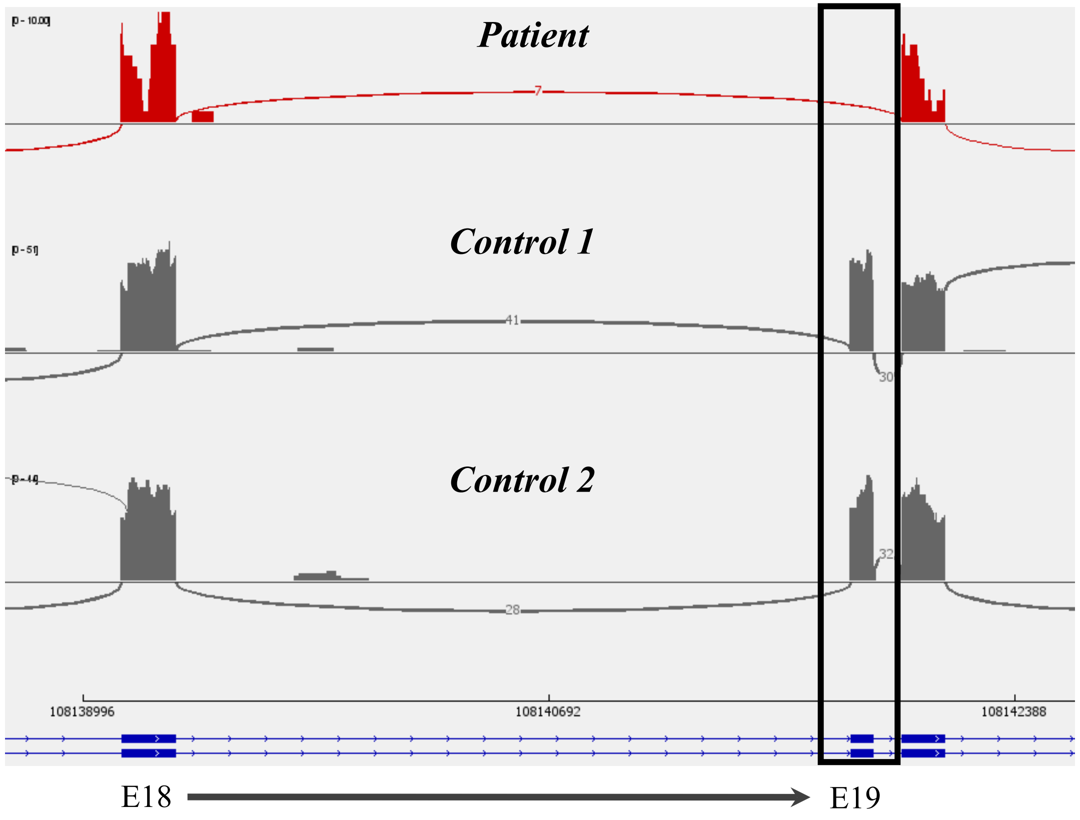

2) Pseudo Exon

***FBXL4* NM_001278716.1:c.1104-2646A>G**

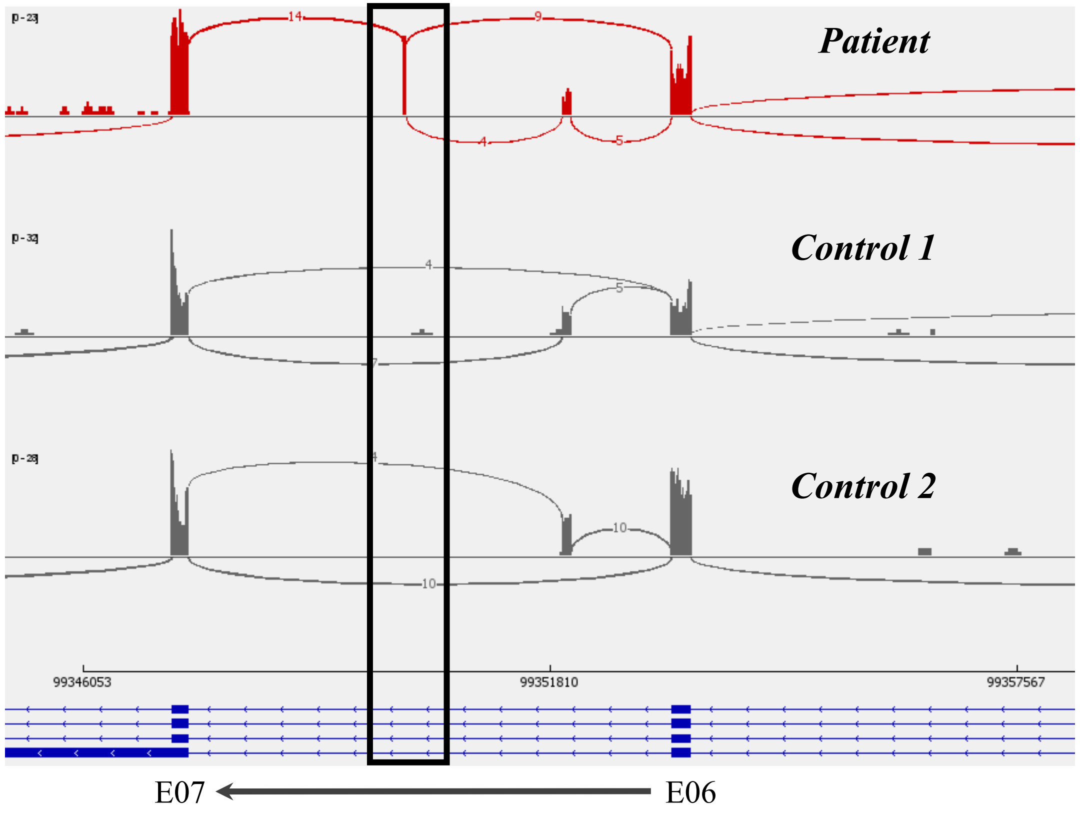

Supplementary Figure 4. Screenshots from the Integrated Genome viewer (IGV) showing splicing alterations from the examples shown in Figure 4.

3) Cryptic 5'splice site with intron retention
COQ2 **NM_015697.7:c.403+355C>G**

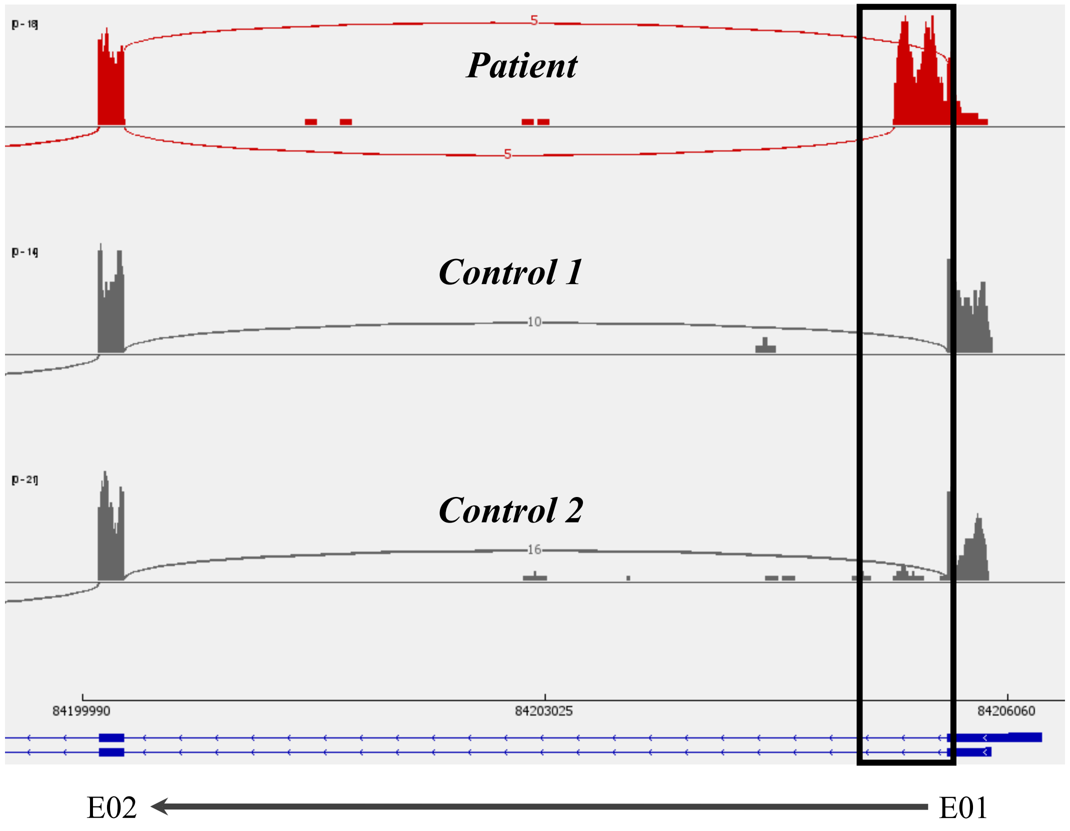

4) Cryptic 3'splice site with intron retention
VPS16 **NM_022575.3:c.1368-11G>A**

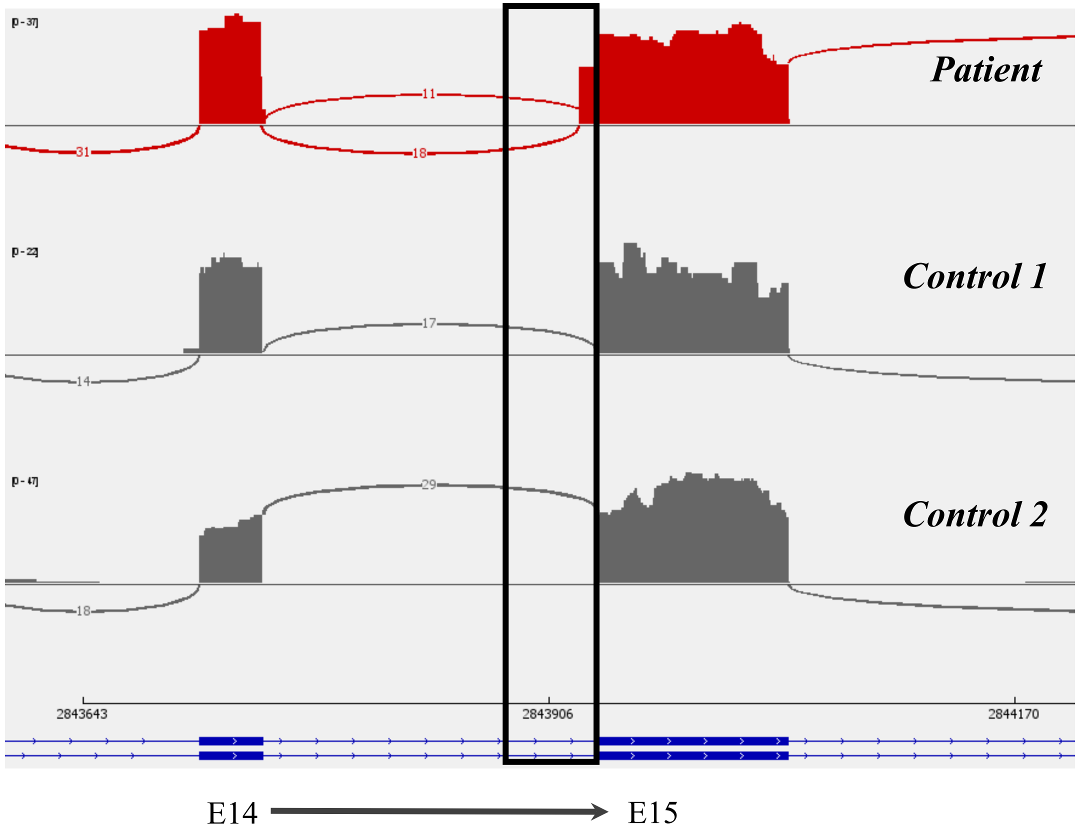

Supplement: Supplementary file 1 — Supplemental Figures [file 41431_2025_1792_MOESM1_ESM.pdf]
