# Supplementary material for: Beyond genomics: using RNA-seq from dried blood spots to unlock the clinical relevance of splicing variation in a diagnostic setting
Source: Eur J Hum Genet. 2025 Jan 28;33(5):614–23. doi: 10.1038/s41431-025-01792-2 (PMC12048715; doi:10.1038/s41431-025-01792-2)
Supplement: Supplementary file 2 — Supplemental material [file 41431_2025_1792_MOESM2_ESM.docx]

**Supplementary table: List of variants relevant to the patient phenotype, including variant classification and RNA-seq result.**

| **Patient sex** | **Variant classification** | **Zygosity** | **Gene** | **SNV nt position** | **Intron** | **RNA-seq result** | **Effect on mRNA** | **Splice AI** |
| --- | --- | --- | --- | --- | --- | --- | --- | --- |
| **Male** | Likely pathogenic | Homozygous | *ADPRS (ADPRHL2)* | c.517-2_557del | Intron 3 -Exon 4 | Confirmed abnormal splicing | Cryptic donor splice site with intron retention | 1.00 |
| **Male** | Pathogenic | Homozygous | *ATM* | c.2921+3A>T | Intron 19 | Confirmed abnormal splicing | Exon 19 skipped | 0.90 |
| **Male** | Likely Pathogenic | Heterozygous | *COL6A2* | c.736-3C>G | Intron 4 | Unclear | - | 0.77 |
| **Female** | VUS | Homozygous | *COQ2* | c.403+355C>G | Intron 1 | Confirmed abnormal splicing | Cryptic donor splice site with intron retention | 0.63 |
| **Female** | VUS | Heterozygous | *CSNK2B* | c.558-1G>A | Intron 6 | Confirmed abnormal splicing | Loss of splice site with intron retention | 0.98 |
| **Female** | Likely pathogenic | Homozygous | *CSPP1* | c.3141+1G>T | intron 25 | Confirmed abnormal splicing | Exon 25 skipped | 0.99 |
| **Male** | Likely pathogenic | Heterozygous | *CSPP1* | c.3141+1G>T | Intron 25 | Not analyzable (degraded) | - | 0.99 |
| **Female** | VUS | Homozygous | *CTNS* | c.681+5G>A | Intron 9 | Not analyzable (degraded) | - | 0.49 |
| **Female** | VUS | Heterozygous | *CUL3* | c.282+2T>G | Intron 2 | Confirmed abnormal splicing | Cryptic donor splice site with partial exon exclusion | 0.99 |
| **Male** | VUS | Heterozygous | *CYP2R1* | c.1331-1G>A | Intron 4 | Confirmed abnormal splicing | Cryptic acceptor splice site with intron retention | 0.90 |
| **Male** | VUS | Heterozygous | *DEAF1* | c.1127-2A>G | Intron 8 | Confirmed abnormal splicing | Exon 9 skipped, cryptic acceptor splice site with partial exon exclusion | 0.99 |
| **Male** | Likely pathogenic | Homozygous | *DNAJB2* | c.176-2A>G | Intron 3 | Confirmed abnormal splicing | Exon 4 skipped | 0.99 |
| **Female** | Likely pathogenic | Heterozygous | *DYRK1A* | c.1098+2T>C | Intron 7 | Confirmed abnormal splicing | Exon 7 skipped | 0.91 |
| **Female** | Pathogenic | Heterozygous | *DYSF* | c.5785-824C>T | Intron 51 | Confirmed abnormal splicing | Pseudoexon | 0.67 |
| **Male** | VUS | Heterozygous | *EFTUD2* | c.105+2T>A | Intron 2 | Not analyzable (low coverage) | - | 1.00 |
| **Female** | Likely pathogenic | Heterozygous | *EFTUD2* | c.2466+2T>C | Intron 24 | Confirmed abnormal splicing | Exon 24 skipped | 0.91 |
| **Female** | VUS for both variants | Heterozygous | *EXOSC3* | c.474+110G>A, c.619_622dup | Intron 2 | No Effect | - | 0.00,  0.02 |
| **Female** | VUS | Homozygous | *FBXL4* | c.1104-2646A>G | Intron 6 | Confirmed abnormal splicing | Pseudoexon | 0.95 |
| **Female** | Pathogenic | Heterozygous | *FOXP1* | c.1771-3C>T | Intron 19 | Confirmed abnormal splicing | Exon 20 skipped | 0.21 |
| **Male** | Likely Pathogenic | Homozygous | *FUCA1* | c.970-1G>A | Intron 5 | Not analyzable (degraded) | - | 1.00 |
| **Female** | Pathogenic | Homozygous | *GAA* | c.2647-7G>A | Intron 18 | Confirmed abnormal splicing | Intron retention | 1.00 |
| **Female** | Risk factor | Homozygous | *GALT* | c.378-27G>C | Intron 4 | Confirmed abnormal splicing | Intron retention | 0.01 |
| **Female** | Risk factor | Homozygous | *GALT* | c.507+62G>A | Intron 5 | Confirmed abnormal splicing | Cryptic acceptor splice site with intron retention | 0.13 |
| **Female** | Risk factor | Homozygous | *GALT* | c.508-24G>A | Intron 5 | Confirmed abnormal splicing | Intron retention | 0.16 |
| **Female** | Risk factor | Homozygous | *GALT* | c.-129_-126del | Upstream | Unclear | - | N/A |
| **Male** | Likely pathogenic | hemizygous | *GLA* | c.801+48T>G | Intron 5 | Confirmed abnormal splicing | Cryptic acceptor splice site with intron retention | 0.16 |
| **Female** | Pathogenic | Homozygous | *HBA2* | c.*94A>G | 5'UTR | Confirmed abnormal splicing | Multiple splicing abnormalities | 0.00 |
| **Female** | Pathogenic | Homozygous | *HBA2* | c.*93_*94del | 5' UTR | Confirmed abnormal splicing | Multiple splicing abnormalities | 0.00 |
| **Female** | Pathogenic | Heterozygous | *HBB* | c.92+6T>C | Intron 2 | Confirmed abnormal splicing | Cryptic donor splice site with partial exon exclusion | 0.45 |
| **Female** | Pathogenic | Heterozygous | *HBB* | c.315+1G>A | Intron 2 | Confirmed abnormal splicing | Exon 2 skipped, cryptic donor splice site with intron retention | 1.00 |
| **Male** | VUS | Hemizygous | *HDAC8* | c.165-58del | Intron 2 | Unclear | - | 0.03 |
| **Female** | Likely Pathogenic | Homozygous | *HEXA* | c.492+3_492+6del | Intron 4 | Confirmed abnormal splicing | Exon 4 skipped | 0.48 |
| **Female** | Likely pathogenic | Homozygous | *HPS1* | c.988-44_1016del | Exon 12 | Confirmed abnormal splicing | Splice site loss | 0.99 |
| **Male** | VUS | Hemizygous | *KDM5C* | c.2243+5G>A | Intron 15 | Confirmed abnormal splicing | Cryptic donor splice site with intron retention, exon 16 skipped | 0.37 |
| **Male** | VUS | Heterozygous | *KMT2A* | c.5005-1_5005del | Exon 16 | Not analyzable (degraded) | - | 0.99 |
| **Female** | Likely Pathogenic | Homozygous | *LARP7* | c.528_574-12del | Partially encompassing exon 5 | Not analyzable (low coverage) | - | 1.00 |
| **Male** | VUS | Heterozygous | *LEMD3* | c.2573-11T>G | Intron 12 | Confirmed abnormal splicing | Cryptic acceptor splice site with intron retention | 0.94 |
| **Male** | Likely pathogenic | Heterozygous | *LMNA* | c.1968+2T>C | Intron 11 | Confirmed abnormal splicing | Cryptic donor splice site with partial exon exclusion (alternative transcript) | 0.56 |
| **Female** | VUS | Heterozygous | *LZTR1* | c.1942+2T>C | Intron 16 | Confirmed abnormal splicing | Cryptic donor splice site with exon exclusion | 0.82 |
| **Male** | VUS | Heterozygous | *MANBA* | c.177+2T>C | Intron 1 | Confirmed abnormal splicing | Intron retention | 0.94 |
| **Male** | Likely pathogenic | Heterozygous | *MUTYH* | c.779_789-10del | Exon 9 | Not analyzable (degraded) | - | 0.99 |
| **Male** | VUS | Homozygous | *NCF1* | c.575-6G>A | Intron 6 | No Effect | - | 0.61 |
| **Male** | VUS | Homozygous | *NDUFS2* | c.703-11T>G | Intron 7 | Confirmed abnormal splicing | Exon 8 skipped | 0.29 |
| **Female** | VUS | Heterozygous | *NF1* | c.586+5G>C | Intron 5 | Not analyzable (low coverage) | - | 0.94 |
| **Female** | VUS | Heterozygous | *NF1* | c.6820-1G>C | Intron 45 | Confirmed abnormal splicing | Exon 45 skipped | 0.98 |
| **Female** | Pathogenic | Heterozygous | *NF1* | c.6704+1del | Exon 44 | Confirmed abnormal splicing | Two cryptic donor splice sites with intron retention | 1.00 |
| **Male** | VUS | Homozygous | *NFU1* | c.-227G>A | 5'UTR | Not analyzable | - | N/A |
| **Male** | Likely pathogenic | Heterozygous | *NIPBL* | c.4422-2A>T | Intron 20 | Confirmed abnormal splicing | Exon 21 skipped | 0.99 |
| **Female** | Likely pathogenic | Homozygous | *NPC1* | c.2130+1G>A | Intron13 | Confirmed abnormal splicing | Cryptic donor splice site with intron retention | 0.98 |
| **Female** | VUS | Homozygous | *PIDD1* | c.2042-2A>G | Intron 12 | Confirmed abnormal splicing | Intron retention | 0.99 |
| **Female** | Likely pathogenic | Heterozygous | *PLA2G6* | c.1349-1G>A | Intron 9 | Unclear | - | 0.99 |
| **Male** | Likely pathogenic | Heterozygous | *PNKP* | c.1029+2T>C | Intron 11 | Confirmed abnormal splicing | Exon 11 skipped | 0.93 |
| **Female** | VUS | Heterozygous | *PRPF31* | c.-349del | 5'UTR | Not analyzable (degraded) | - | 0.02 |
| **Male** | Likely pathogenic | Heterozygous | *PRPF31* | c.856-1G>A | Intron 8 | Confirmed abnormal splicing | Cryptic acceptor splice site with intron retention | 0.98 |
| **Female** | Likely pathogenic | Heterozygous | *PTEN* | c.209+5G>A | Intron 3 | Confirmed abnormal splicing | Exon 4 skipped | 0.99 |
| **Male** | Pathogenic | Homozygous | *RBM8A* | c.-21G>A | 5'UTR | No Effect | - | 0.00 |
| **Male** | VUS | Hemizygous | *RPGR* | c.1246-17A>G | Intron 10 | Not analyzable  (degraded) | - | 0.51 |
| **Male** | Pathogenic | Hemizygous | *SLC6A8* | c.1392+24_1393-30del | Intron 9 | Confirmed abnormal splicing | Exon 9 skipped | 0.63 |
| **Female** | VUS | Heterozygous | *SMARCA4* | c.1594-541G>A | Intron 9 | No Effect | - | 0.00 |
| **Female** | Likely Pathogenic | Heterozygous | *SMARCB1* | c.1145+1G>A | Intron 8 | Confirmed abnormal splicing | Two cryptic donor splice sites (GC and GU) with intron retention, exon 8 skipped | 0.01 |
| **Male** | Likely pathogenic | Heterozygous | *SMG9* | c.701+4A>G | Intron 6 | Not analyzable (low coverage) | - | 0.91 |
| **Male** | VUS | Homozygous | *SMN1* | c.834+1692C>G | Intron 7 | No Effect | - | 0.00 |
| **Male** | Pathogenic | Homozygous | *SZT2* | c.7702+1G>T | Intron55 | Not analyzable (degraded) | - | 1.00 |
| **Male** | VUS | Heterozygous | *TNRC6B* | c.46-2A>G | Intron 3 | Confirmed abnormal splicing | Intron retention | 0.01 |
| **Female** | VUS | Heterozygous | *VPS16* | c.1368-11G>A | Intron 23 | Confirmed abnormal splicing | Cryptic acceptor splice site with intron retention | 1.00 |
| **Female** | VUS | Heterozygous | *VPS16* | c.2375+1G>T | Intron 23 | Confirmed abnormal splicing | Intron retention, cryptic splice site with partial exon exclusion | 0.98 |
| **Female** | VUS | Heterozygous | *VPS16* | c.1475A>G | Intron 14 | Confirmed abnormal splicing | Exon 15 skipped | 0.82 |
| **Male** | Likely pathogenic | Hemizygous | *WAS* | c.777+5G>C | Intron 8 | Confirmed abnormal splicing | Exon 8 skipped | 0.95 |

N/A: Not available
